# Regulatory mechanisms of betacellulin in CXCL8 production from lung cancer cells

**DOI:** 10.1186/1479-5876-12-70

**Published:** 2014-03-16

**Authors:** Lin Shi, Lingyan Wang, Beibei Wang, Sanda Maria Cretoiu, Qun Wang, Xiangdong Wang, Chengshui Chen

**Affiliations:** 1 Department of Pulmonary Medicine, The First affiliated Hospital, Wenzhou Medical University, Wenzhou, China; 2 Biomedical Research Center, Zhongshan Hospital, Fudan University, Shanghai, China; 3 Department of Thoracic Surgery, Zhongshan Hospital, Fudan University, Shanghai, China; 4 Department of Pulmonary Medicine, Zhongshan Hospital, Fudan University, Shanghai, China; 5 Division of Cellular and Molecular Medicine, Department of Morphological Sciences, Faculty of Medicine, Carol Davila University of Medicine and Pharmacy, Bucharest, Romania

**Keywords:** Lung cancer, Betacellulin, Interleukin-8, EGFR, PI3K

## Abstract

**Background:**

Betacellulin (BTC), a member of the epidermal growth factor (EGF) family, binds and activates ErbB1 and ErbB4 homodimers. BTC was expressed in tumors and involved in tumor growth progression. CXCL8 (interleukin-8) was involved in tumor cell proliferation via the transactivation of the epidermal growth factor receptor (EGFR).

**Materials and methods:**

The present study was designed to investigate the possible interrelation between BTC and CXCL8 in human lung cancer cells (A549) and demonstrated the mechanisms of intracellular signals in the regulation of both functions. Bio-behaviors of A549 were assessed using Cell-IQ Alive Image Monitoring System.

**Results:**

We found that BTC significantly increased the production of CXCL8 through the activation of the EGFR-PI3K/Akt-Erk signal pathway. BTC induced the resistance of human lung cancer cells to TNF-α/CHX-induced apoptosis. Treatments with PI3K inhibitors, Erk1/2 inhibitor, or Erlotinib significantly inhibited BTC-induced CXCL8 production and cell proliferation and movement.

**Conclusion:**

Our data indicated that CXCL8 production from lung cancer cells could be initiated by an autocrine mechanism or external sources of BTC through the EGFR–PI3K–Akt–Erk pathway to the formation of inflammatory microenvironment. BTC may act as a potential target to monitor and improve the development of lung cancer inflammation.

## Background

The epidermal growth factor receptor (EGFR) consists of an extracellular ligand-binding domain, a transmembrane domain and an intracellular tail with an ATP-binding site, tyrosine kinase activity, and capability of autophosphorylation [1,2]. EGFR has been found to contribute the lung development [3] and multiplicity of cancer-related signal transduction pathways like cellular proliferation, adhesion, migration, neoangiogenesis, and apoptosis inhibition [4]. EGFR is also responsible for the sensitivity of human non-small cell lung cancer (NSCLC) cells to therapies and prognosis of patients [5,6]. There are numerous ligands to bind with EGFR, such as EGF, transforming growth factor-α (TGF-α), heparin-binding EGF-like growth factor (HB-EGF), epiregulin (ER), amphiregulin (AR), neuregulin (NRG) subfamily and betacellulin (BTC) [7]. The activation of EGFR can initiate the downstream signaling cascades, e.g. the Ras/mitogen-activated protein kinase (MAPK) and phosphoinositide-3 kinase (PI3K)/Akt [8-11].

BTC is a member of EGF family and acts as a potent mitogen for cell types, with the higher affinity and specificity for ErbB1/EGFR and ErbB4. Homologous or heterologous dimers of ErbB family receptor are then formed to activate signal transduction pathways, such as PI3K/PDK1/Akt and RAS/RAF/MEK/Erk, leading to a series of biological effects [12,13]. Abnormal phosphorylation of Akt and Erk1/2 was considered as an important factor in the prognosis of cancer [14] and constitutive activation of EGFR–Akt–mTOR was found in about 18% of NSCLCs [15].

Our previous study on disease-specific biomarkers of patients with acute exacerbations of chronic obstructive pulmonary disease (AECOPD) by integrating inflammatory mediators with clinical informatics demonstrated that BTC played an important role in the occurrence of AECOPD and was associated with the disease severities [16]. We also found that EGFR–PI3K–Akt–Erk pathway was involved in the development of lung cancer inflammatory microenvironment by the hyper-production of CXCL8 [17], responsible for leukocyte recruitment, cancer proliferation, and angiogenesis [18]. The present study further aimed at understanding the potential association and interaction mechanisms between BTC and CXCL8 in the inflammatory microenvironment, exploring the expression and biological function of BTC gene and protein and its receptors in human lung cancer cells, and define the role of BTC in the regulation of CXCL8 expression and production in lung cancer. The present study furthermore investigated the involvement of EGFR–PI3K–Akt–Erk activation in CXCL8 production induced by BTC with consequences on lung cancer cell proliferation and movement.

## Materials and methods

### Cell lines and reagents

Human lung cancer cell line A549 cells were cultured in RPMI 1640 supplemented with penicillin (100 U/ml), streptomycin (100 mg/ml), and 10% heat inactivated fetal bovine serum (FBS). Human recombinant BTC, Enzyme-Linked Immunosorbent Assay (ELISA) kits for CXCL8, anti-human BTC neutralizing antibody were purchased from R&D Systems (Shanghai, China). PI3K/mTOR inhibitors (BEZ235, GDC0941, SHBM1009) and Erk1/2 inhibitor (PD98059) were purchased from Biovision (California, USA). EGFR inhibitor (Erlotinib) was from Roche (Basel, Switzerland). EGFR, ErbB2, ErbB3 and ErbB4 antibodies for immunofluorescent staining were purchased from Abcam (Hong Kong, China). Cell-IQ live cell imaging platform was manufactured by Chipmantech (Tampere, Finland) and equipped in Center for Biomedical Research, Zhongshan Hospital, Fudan University, Shanghai, China.

### Measurement of gene expression

Total RNA was isolated using a guanidinium isothiocyanate/chloroform based technique (TRIZOL, Invitrogen, USA) and measured with OD 260 nm. RNA was subsequently reversed and transcribed to cDNA with the SuperScript First-strand Synthesis System (Invitrogen, USA). Quantitative RT-PCR was carried out using an ABI 7000 PCR instrument (Eppendorf, Hamburg, Germany) with the two-stage program parameters, as follows: 1 min at 95°C, and then 40 cycles of 5 s at 95°C and 30s at 60°C. The sequences of the primer sets used for this analysis are as follows: BTC, 5′-TGAAACTAATGGCCTCCTCTGT -3′ (forward [F]) and 5′-CTTTTACGACGTTTCCGAAGAG -3′ (reverse [R]); CXCL8, 5′-TTGCCAAGGAGTGCTAAAGAA -3′ (F) and 5′-GCCCTCTTCAAAAACTTCTCC -3′ (R); and for human glyceraldehyde-3-phosphate dehydrogenase (GAPDH), 5′-CCACCCATGGCAAATTCCATGGCA-3′ (F) and 5′-TCTACACGGCAGGTCAGGTCCACC-3′ (R). Specificity of the produced amplification product was confirmed by examination of dissociation reaction plots. Each sample was tested in triplicate with quantitative RT-PCR, and each group had six wells.

### Production of BTC and CXCL8

A549 cells were cultured in 24 well cell culture microplates at 1 × 10^5^ cells/well for 24 h and then treated with lipopolysaccharide (LPS) (Escherichia coli, 055:B5, Sigma, St. Louis, MO) at concentrations of 0.01, 0.1, and 1 μg/ml for an additional 24 h, respectively, to study LPS-induced productions of BTC or CXCL8. Cells were pre-incubated with an anti-human BTC neutralizing antibody at concentrations of 1, 10, 100 ng/ml or IgG as non-specific control 2 h before LPS stimulation to study the role of BTC in LPS-induced CXCL8 production. Cells were treated with BTC at 0.1 μg/ml or vehicle and pretreated with BEZ235, GDC0941, SHBM1009, Erlotinib, or PD98059 at 0.1, 1, or 10 μM, respectively, for 24 h to investigate the involvement of various signal pathways. Each experiment was done in six replicate wells for each drug concentration and each time point. Levels of BTC and CXCL8 proteins in supernatant were measured by ELISA at the absorbanceof 450 nm.

### Expression of receptors

A549 were fixed with 4% paraformaldehyde, washed thrice, permeabilized with 0.1% Triton X-100, and blocked with 10% goat serum. Cells were incubated overnight with mouse monoclonal antibodies against EGFR, ErbB2, ErbB3, or ErbB4, respectively, and then strained with FITC conjugated anti mouse IgG antibody (Abcam, HK, China, 1:100). The counterstaining was performed with DAPI (4, 6^′^-diamidino-2-phenylindole) and cells were examined under immunofluorescence microscope (Olympus/BX51, Tokyo, Japan).

### Measurements of apoptosis

Apoptosis was analyzed by FACS as previously described [19]. Lung cancer cells A549 (1 × 10^6^ cell/ml) were treated with TNF-α at 20 ng/ml/CHX at 2.5 μmol/l (Sigma, St. Louis, MO) for 24 h in the presence or absence of BTC at 0.01, 0.1, 1 μg/ml, respectively. Cells were then harvested and washed thrice, resuspended in pre-diluted binding buffer, and stained with Annexin V-FITC (Sigma, St. Louis, MO) for 20 min. Cell apoptosis was analyzed by flow cytometry (BD Bioscience, San Diego,CA,USA) using Cell Quest Software (Biomedika, Canada).

### Measurements of cell bio-behaviors

The cell bio-behaviors including total cell number, cell differentiation, and cell movement were measured by the real-time cell monitoring system, using a Cell-IQ cell culturing platform (Chip-Man Technologies, Tampere, Finland), equipped with a phase-contrast microscope (Nikon CFI Achromat phase contrast objective with 10x magnification) and a camera (Nikon, Japan). The equipment was controlled by Imagen software (Chip-Man Technologies). Images were captured at 5 min intervals for 72 h. Analysis was carried out with a freely distributed Image software (Cell-IQ Imagen v2.9.5c, McMaster Biophotonics Facility, Hamilton, ON), using the Manual Tracking plug-in created by Fabrice Cordelieres (Institut Curie, Orsay, France). Cell-IQ system uses machine vision technology to monitor and record time-lapse data, and it can also analyze and quantify cell functions and morphological parameters. The movement of each individual cell was measured in the image field by metering the distance of cell movement.

### Statistical analysis

Data were represented as mean ± SEM. Statistical significance was compared between groups by the Student’s *t*-test, after ANOVA analyses. Increased rates of total cell number and differentiation were calculated as the following: Rate (%) = (value at each time point-value of primary seeding cells)/value of primary seeding cells × 100. Cell movement was calculated as the mean of the distance of every cell moving between two images (5 min interval). *p*-Values less than 0.05 was considered to be significant.

## Results

**Figure 1:**
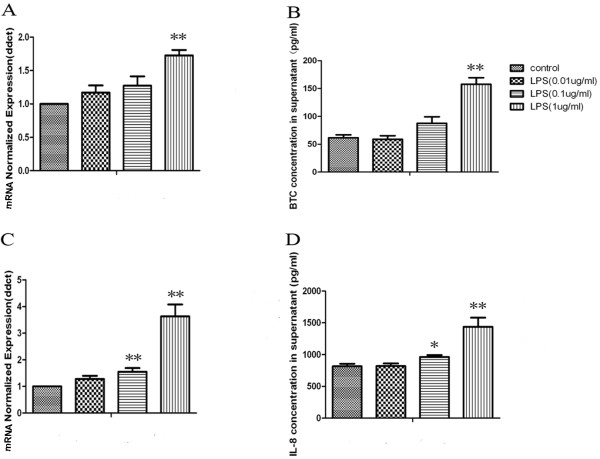
**LPS induces increased production of BTC and IL-8 in human lung cancer cells A549.** A549 cells grown in complete medium were left untreated (control) or treated with LPS (0.01, 0.1 and 1 μg/ml) for 4 h. Total RNA was extracted and subjected to reverse transcription followed by qPCR to detect BTC **(A)** and IL-8 **(C)** mRNA, as described in Material and Methods. Data were normalized to control. A549 cells (1 × 10^5^/ml) were stimulated with LPS for 24 h, then the cytokines including BTC **(B)** and IL-8 **(D)** in cell-free supernatants were assayed using sandwich ELISA. Each data point represents mean ± SEM of three experiments. * and ** stand for *P* -values less than 0.05 and 0.01, as compared to control.

**Figure 2:**
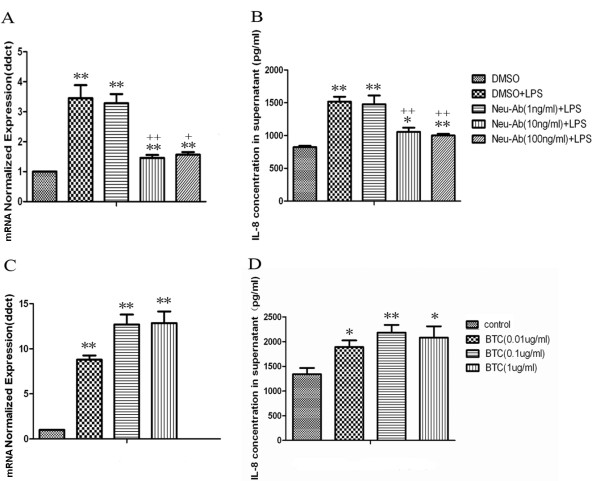
**LPS-induced IL-8 production is BTC dependent in human lung cancer cells A549.** A549 cells were pretreated with anti-BTC neutralizing antibody (Neu-Ab) for 2 h. Subsequently, cells were stimulated with LPS (1 μg/ml) for 4 h. Total RNA was extracted and subjected to reverse transcription followed by qPCR to detect IL-8 mRNA **(A)**. IL-8 in cell-free supernatants after 24 h stimulated by LPS were assayed using sandwich ELISA **(B)**. A549 cells were then stimulated with BTC (0.01, 0.1 and 1 μg/ml) for 4 h to detect IL-8 mRNA **(C)** or 24 h to detect its secretion of IL-8 in supernatants **(D)**. Each data point represents mean ± SEM of three experiments. * and ** stand for *P-* values less than 0.05 and 0.01, in comparison with untreated control cells, and + and ++ stand for *P -*values less than 0.05 and 0.01, as compared to LPS (1 μg/ml) and DMSO, respectively.

The mRNA expression and protein production of BTC in A549 cells significantly increased at the stimulation of LPS at 1 μg/ml, while those of CXCL8 significantly increased at both 0.1 and 1 μg/ml with a dose-dependent parttern, as shown in Figure 1 (p < 0.05 or 0.01, respectively). A positive correlation of BTC and CXCL8 expression in lung cancer was observed. Cells were pretreated with anti-human BTC neutralizing antibody to investigate the potential role of endogenous BTC in LPS-induced over-expression and over-production of CXCL8 mRNA and proteins. Pretreatment with BTC neutralizing antibodies at concentrations of 10 and 100 ng/ml could significantly prevent from LPS-induced over-expression of CXCL8 mRNA and over-production of CXCL8 proteins, as compared with those pretreated with vehicle and challenged with LPS (p < 0.05 or 0.01, respectively, in Figure 2A and B). The stimulation of exogenous BTC proteins from the dose of 0.01 μg/ml and on significantly increased the expression and production of CXCL8 mRNA and proteins (p < 0.05 or 0.01 in Figure 2C and D), as compared with those stimulated with vehicle.

**Figure 3:**
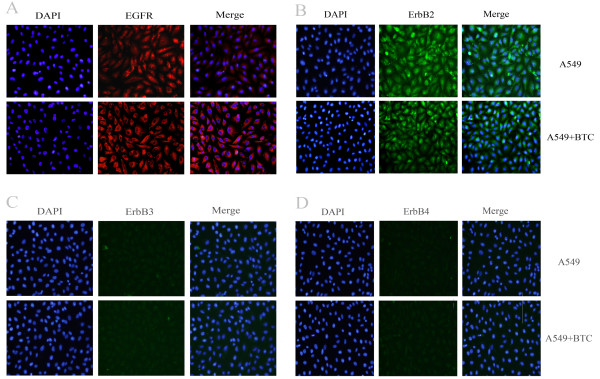
**Expression of ErbB family receptors in A549 human lung tumor cell line.** A549 cell line is positive for EGFR (red labeling) **(A)**, and ErbB2 (green) **(B)** and negative for ErbB3 **(C)** and ErbB4 **(D)** (FITC staining, green), respectively, in immunofluorescence analysis. DAPI staining (blue) indicates the localization of nuclei. One representative image from three independent experiments is presented.

**Figure 4:**
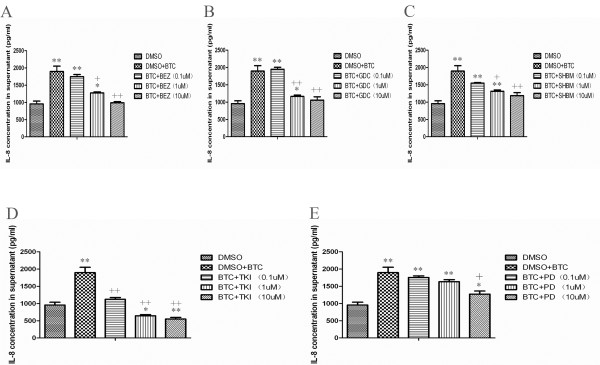
**Treatment with PI3K inhibitors, Erk1/2 inhibitor or Erlotinib significantly inhibited BTC-induced IL-8 production in A549 lung cancer cells.** IL-8 production from cells was measured 24 h after the culture with DMSO alone, BTC at 0.1ug/ml plus DMSO, BEZ235 (BEZ; **A**), GDC0941 (GDC; **B**), SHBM1009 (SHBM; **C**) or Erlotinib (TKI; **D**), PD98059 (PD; **E**) at doses of 0.1, 1.0 or 10 μM. * and ** stand for *P-* values less than 0.05 and 0.01 as compared to cells only with DMSO, and + and ++ stand for *P*-values less than 0.05 and 0.01, as compared to BTC and DMSO, respectively. Data is presented as mean ± SEM and each group has six measurements.

Figure 3 demonstrated the expression of ErbB1/EGFR, ErbB2, ErbB3, or ErbB4 on A549 cells evaluated by immunofluorescence staining. We found that A549 cells constitutively expressed EGFR (Figure 3A) and ErbB2 (Figure 3B), rather than ErbB3 (Figure 3C) and ErbB4 (Figure 3D), of which the expression of EGFR increased 24 hours after the stimulation of exogenous BTC. Our pilot study demonstrated that BTC at 0.1 μg/ml could significantly increase production of CXCL8 A549 cells from 12 h and on, which maintained till at 48 h. Treatments with PI3K inhibitors (BEZ235, GDC0941 and SHBM1009) at 1 μM, 10 μM and Erk1/2 inhibitor (PD98059) at 10 μM significantly inhibited BTC-induced CXCL8 production, as compared to cells treated with vehicle (*P* < 0.05 or 0.01, respectively, Figure 4A-E). Treatment with Erlotinib (TKI) at all concentrations significantly prevented BTC-stimulated over-production of CXCL8 (*P* < 0.01, Figure 4D).

**Figure 5:**
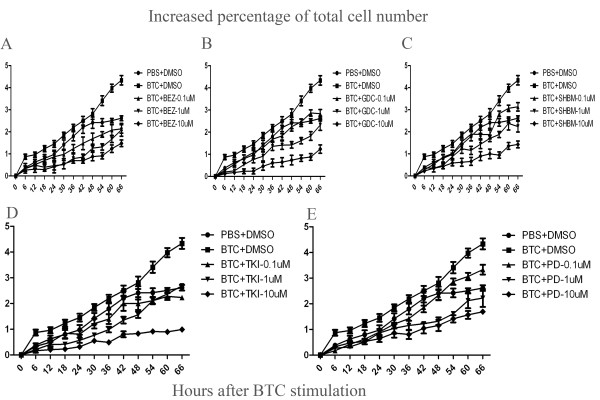
**The increased percentage of the total number of lung cancer cells (A549 cells) was assessed using Cell-IQ Alive Image Monitoring System, at 72 h.** The percentage of total number of cells significantly increased (∎) after the stimulation with BTC, compared to the average of total cells values treated with vehicle (DMSO) alone (●), while cells treated with BEZ235 **(A)**, GDC0941 **(B)**, SHBM1009 **(C)**, Erlotinib **(D)** or PD98059 **(E)** showed a lower increase in the percentage of total number of cells, at doses of 0.1, 1 or 10 μM, respectively. Data were presented as mean ± SEM and each group has 6–12 measurements.

**Figure 6:**
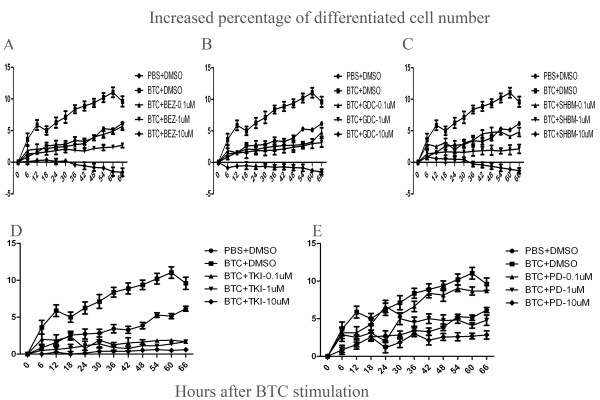
**The percentage of the differentiated lung cancer cells (A549 cells) was assessed using Cell-IQ Alive Image Monitoring System, at 72 h.** The percentage of differentiated cells significantly increased (∎) after the stimulation with BTC, compared to the average of total cells values treated with vehicle (DMSO) alone (●), while cells treated with BEZ235 **(A)**, GDC0941 **(B)**, SHBM1009 **(C)**, Erlotinib **(D)** or PD98059 **(E)** showed a lower increase, or even a decrease in the percentage of differentiated cells, at doses of 0.1, 1 or 10 μM, respectively. Data were presented as mean ± SEM and each group has 6–12 measurements.

**Figure 7:**
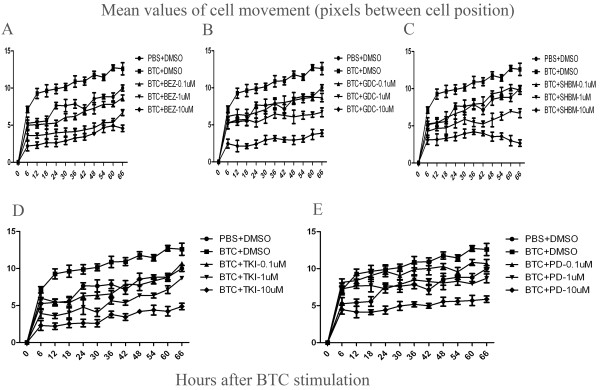
**The percentage of lung cancer cells (A549 cells) movement was assessed using Cell-IQ Alive Image Monitoring System, at 72 h.** The percentage of A549 cells movement significantly increased (∎) after the stimulation with BTC, compared to the average of total cells values treated with vehicle (DMSO) alone (●), while cells treated with BEZ235 **(A)**, GDC0941 **(B)**, SHBM1009 **(C)**, Erlotinib **(D)** or PD98059 **(E)** showed a lower increase, or even a decrease in the percentage of A549 cells movement, at doses of 0.1, 1 or 10 μM, respectively. Data were presented as mean ± SEM and each group has 6–12 measurements.

**Figure 8:**
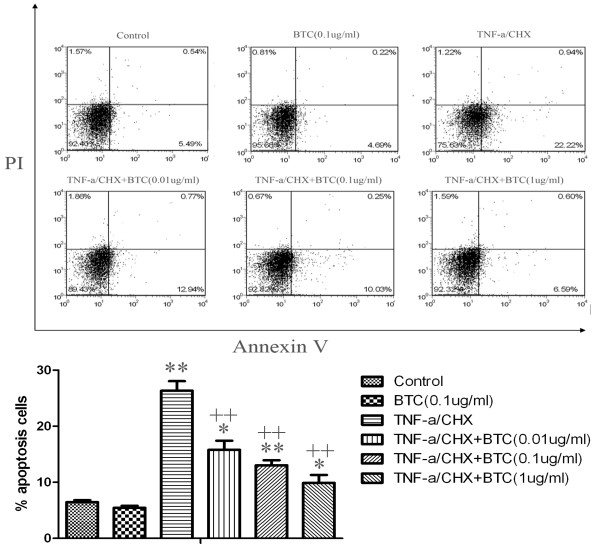
**BTC pretreatment induces resistance of human lung cancer cells to apoptosis induction by TNF-α/CHX.** A549 cells (1 × 10^6^/ml) were pretreated with BTC for 4 h, then stimulated with 20 ng/ml TNF-α/CHX for 24 h. The cells were stained with annexin V and PI and subjected to FACS assay of cellular apoptosis. ** stand for *P-* value less than 0.01 as compared to control (DMSO only), and ++ stand for *P*-value less than 0.01, as compared to BTC + DMSO. Data were presented as mean ± SEM of three independent experiments.

The percentage of total cell number significantly increased after the stimulation of BTC at 0.1 μg/ml, as compared to those stimulated with vehicle ( *P* < 0.05 or 0.01, respectively, Figure 5), which was significantly inhibited by BEZ235 (Figure 5A), GDC0941 (Figure 5B), SHBM1009 (Figure 5C), Erlotinib (Figure 5D) or PD98059 (Figure 5E) at various concentrations. Of them inhibitory effects of PI3K inhibitors (BEZ235, GDC0941 and SHBM1009) showed a dose-dependent pattern. Inhibitors also significantly inhibited BTC-increased percentages of differentiated cells, as shown in Figure 6A–E. Figure 7A–E demonstrated similar inhibitory effects of inhibitors on the BTC-increased cell movements, as compared to BTC stimulation alone (*P* < 0.05 or 0.01, respectively). The number of apoptotic cells significantly increased in cells pretreated with vehicle and stimulated with TNF-α/CHX for 24 h, as compared with those pretreated with vehicle or BTC without the stimulation (p < 0.01, respectively, Figure 8). Cells pretreated with different doses of exogenous BTC developed into apoptosis less than those pretreated with vehicle after the stimulation with TNF-α/CHX.

## Discussion

BTC is expressed in bronchial mucosa and lung tissue cells, e.g. the alveolar and airway epitheliums, fibroblasts, and macrophages [8,20]. The evidence from our previous studies and others suggested that BTC play a critical role in the development of lung inflammation through the regulation of the cytokine secretion pattern and tumor cell progression through EGFR ligation, possibly associated with the over-production of CXCL8 [21-25]. The activation of the EGFR pathway could contribute to the over-expression of CXCL8 in human bronchial epithelial cells by multi-stimuli, e.g. HB-EGF [26], MMP-12 [27]. We found that EGF was involved in the development of the lung cancer inflammatory microenvironment through the over-production of CXCL8 associated with the activation of EGFR pathway [17]. The present study provided the further evidence that both BTC and CXCL8 could be over-produced directly by lung cancer cell per se in the inflammatory condition and/or stimuli like LPS.

Our data indicated that lung cancer cells per se may act as a primary receptor to be stimulated and challenged by inflammatory factors and as the secondary reactor to produce the mediators and accelerate the development of the local inflammatory microenvironment. The present study also evidenced that the potential mechanism by which lung cancer cells are regulated to produce chemoattractive factors could be that BTC produced by a lung cancer cell per se or by other neighbor cells might regulate the over-production of secondary inflammatory factors like CXCL8 through EGFR–PI3K–Akt–Erk pathway.

Many regulatory factors may contribute to the molecular mechanism by which LPS can stimulate lung cancer cells to produce inflammatory mediators. Results from the present study demonstrated that both endogenous and exogenous BTC could induce the over-production of CXCL8. The finding that levels of CXCL8 in cells blocked with anti-BTC neutralizing antibody and challenged with LPS were still significantly higher than those without LPS indicates the existence of biological efforts from other factors, like EGF [17]. We found that the signal pathway of BTC-EGFR-PI3K axis may play the crucial and dependent role in the mechanism of CXCL8 production of lung cancer cells, evidenced by the finding that the over-production of CXCL8 by BTC was fully prevented by PI3K and EGFR inhibitors. It implies that the BTC-EGFR-PI3K-CXCL8 chain can be the potential of new anti-inflammatory therapeutic target in lung cancer or chronic lung diseases.

The EGFR-dominated signal pathway, e.g. PI3K, Erk1/2 and STAT, are related to CXCL8 expression in airway epithelium cells [28,29]. We provided direct evidence that A549 cells constitutively expressed EGFR and ErbB2, while the expression of EGFR increased after BTC stimulation in human lung cancer cells. PI3K inhibitors (BEZ235, GDC0941 and SHBM1009) and Erk1/2 inhibitor PD98059 could inhibit over-production of CXCL8 initiated by the over activation of BTC-EGFR pathway.

The PI3K activation has been recently found to play the important role in the development of acute and chronic lung inflammation and injury [30]. PI3K/Akt and Erk1/2 pathways could play the decisive role in lung cancer development and proliferation [30,31], while the inhibition of PI3K/Akt pathway could reduce the migration and invasion of NSCLC cells [32]. We found that BTC could increase the proliferation, differentiation and movement of lung cancer cells, which could be down-regulated by PI3K, Erk, and EGFR inhibitors. The pretreatment with BTC could increase the resistance of lung cancer cells against TNF-α/CHX-induced apoptosis in a dose-associated pattern.

## Conclusions

**Figure 9:**
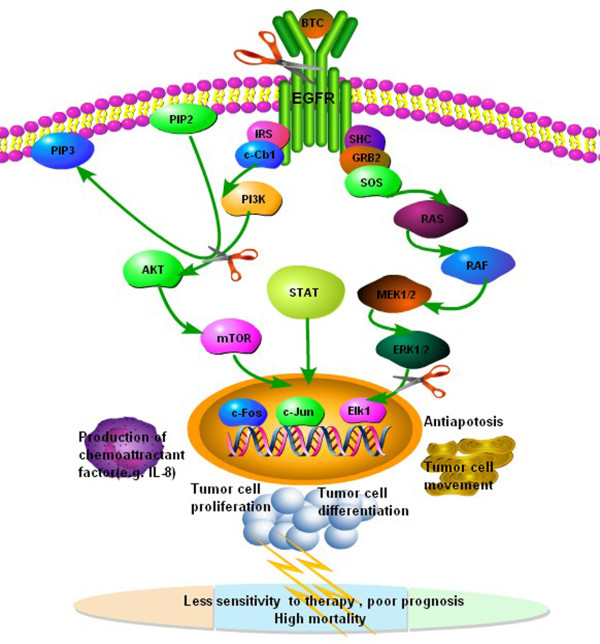
**Proposed mechanism of BTC-stimulated IL-8 production and bio-behaviors of lung cancer cells.** BTC could directly stimulate IL-8 production and bio-behaviors of lung cancer cells through the activation of EGFR, PI3K/Akt, and Erk signal pathway, then lead to the recruitment of inflammatory cells in the cancer tissue and the formation of inflammatory microenvironment. This has been highlighted as an important factor responsible for the sensitivity of human lung cancer to therapies and prognosis of patients.

In summary, the present study demonstrated that LPS increased the over-production of BTC and CXCL8 from human lung cancer cells, which could be blocked by anti-BTC neutralizing antibody. Both endogenous and exogenous BTC could increase the over-production CXCL8 through EGFR–PI3K–Akt–Erk pathway activation. Of EGFRs, EGFR expression increased after the stimulation of BTC. BTC also increased the proliferation, differentiation, and movement of lung cancer cells and increased cell resistance against apoptosis. It indicates that lung cancer cells per se contribute to the development of the local inflammatory microenvironment, probably leading to the recruitment of inflammatory cells in the cancer tissue and the formation of inflammatory microenvironment (Figure 9). Thus, our data indicate that the signal pathway of BTC-EGFR–PI3K–Akt–Erk-CXCL8 plays an important role in the inflammatory microenvironment in lung cancer, as a novel therapeutic approach to lung cancer.

## Abbreviations

AR: Amphiregulin; AECOPD: Acute exacerbations of chronic obstructive pulmonary disease; BTC: Betacellulin; CXCL8: Interleukin-8; ER: Epiregulin; EGFR: Epidermal growth factor receptor; ELISA: Enzyme-linked immunosorbent assay; HB-EGF: Heparin-binding EGF-like growth factor; LPS: Lipopolysaccharide; MAPK: Mitogen activated protein kinase; NRG: Neuregulin; NSCLC: Non-small cell lung cancer; PI3K: Phosphoinositide-3 kinase; TGF-α: Transforming growth factor-α.

## Competing interests

The authors declare that they have no competing of interests.

## Authors’ contributions

Conceived and designed the study: CC, XW and QW; Performed the biological experiments: LS, LW and BW; Statistical analysis: LS. Wrote the paper: LS and SMC. All authors read and proofed the final manuscript.
